# Ethyl 3,5-dimethyl-1*H*-pyrrole-2-carboxyl­ate

**DOI:** 10.1107/S1600536808029929

**Published:** 2008-09-24

**Authors:** Cláudia T. Arranja, Manuela Ramos Silva, Ana Matos Beja, Ana F. P. V. Ferreira, Abílio J. F. N. Sobral

**Affiliations:** a Chemistry Department, University of Coimbra, P-3004-535 Coimbra, Portugal; b CEMDRX, Physics Department, University of Coimbra, P-3004-516 Coimbra, Portugal

## Abstract

In the title compound, C_9_H_13_NO_2_, there are two independent mol­ecules per asymmetric unit. The mol­ecules are very similar and almost planar, with the ethoxy­carbonyl group *anti* to the pyrrole N atom. The two independent mol­ecules are joined into dimeric units by strong hydrogen bonds between NH groups and carbonyl O atoms.

## Related literature

For general background, see: Bonnett (1995 ▶, 2000 ▶). For related structures, see: Paixão *et al.* (2002 ▶), Ramos Silva *et al.* (2002 ▶); Sobral & Rocha Gonsalves (2001 ▶).
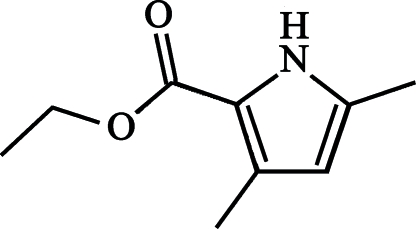


## Experimental

### 

#### Crystal data

C_9_H_13_NO_2_
*M* *_r_* = 167.20
Triclinic, 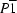

*a* = 8.1357 (2) Å
*b* = 10.5568 (2) Å
*c* = 12.1428 (2) Å
α = 101.5451 (13)°
β = 97.8791 (14)°
γ = 110.4821 (14)°
*V* = 932.52 (4) Å^3^
*Z* = 4
Mo *K*α radiation
μ = 0.08 mm^−1^
*T* = 293 (2) K
0.25 × 0.20 × 0.15 mm

#### Data collection

Bruker APEX CCD area-detector diffractometer
Absorption correction: multi-scan (*SADABS*; Sheldrick, 2000 ▶) *T*_min_ = 0.899, *T*_max_ = 0.987
20370 measured reflections
4456 independent reflections
2368 reflections with *I* > 2σ(*I*)
*R*_int_ = 0.028

#### Refinement

*R*[*F* ^2^ > 2σ(*F* ^2^)] = 0.052
*wR*(*F* ^2^) = 0.182
*S* = 1.03
4456 reflections
223 parameters
H-atom parameters constrained
Δρ_max_ = 0.20 e Å^−3^
Δρ_min_ = −0.21 e Å^−3^

### 

Data collection: *SMART* (Bruker, 2003 ▶); cell refinement: *SAINT* (Bruker, 2003 ▶); data reduction: *SAINT*; program(s) used to solve structure: *SHELXS97* (Sheldrick, 2008 ▶); program(s) used to refine structure: *SHELXL97* (Sheldrick, 2008 ▶); molecular graphics: *ORTEPII* (Johnson, 1976 ▶); software used to prepare material for publication: *SHELXL97*.

## Supplementary Material

Crystal structure: contains datablocks global, I. DOI: 10.1107/S1600536808029929/bt2791sup1.cif

Structure factors: contains datablocks I. DOI: 10.1107/S1600536808029929/bt2791Isup2.hkl

Additional supplementary materials:  crystallographic information; 3D view; checkCIF report

## Figures and Tables

**Table 1:** Hydrogen-bond geometry (Å, °)

*D*—H⋯*A*	*D*—H	H⋯*A*	*D*⋯*A*	*D*—H⋯*A*
N1—H1⋯O4	0.86	2.02	2.857 (2)	166
N2—H2⋯O2	0.86	2.00	2.834 (2)	163

**Fig. 1.**
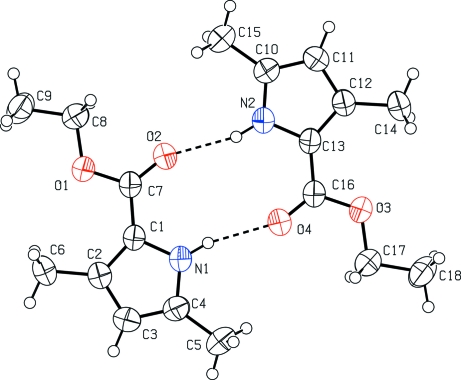
ORTEPII (Johnson, 1976) plot of the title compound. Displacement ellipsoids are drawn at the 50% level. The H-bonds are represented as dashed lines.

**Fig. 2.**
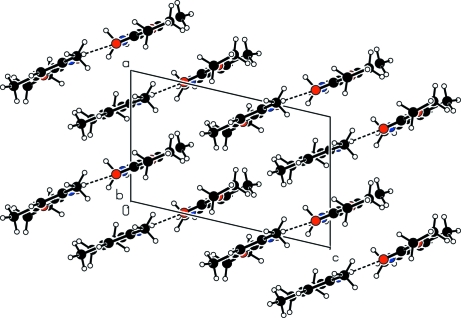
Packing diagram of the title compound.

## supplementary crystallographic information

### Comment

Photodynamic therapy (PDT) is a developing method for the treatment of carcinomas and sarcomas. It consists in a selective absorption of a photosensitizer in a tumor, followed by irradiation with light of a selected wavelength, originating tumor necrosis. The fewer side effects of this therapeutic method when compared to chemotherapy and radiotherapy have prompted the search for new and more efficient photosensitizers, namely porphyrins (Bonnett, 1995, 2000). Pyrroles are building blocks for the synthesis of porphyrins and following our previous structural studies on pyrrole chemistry (Sobral & Rocha Gonsalves, 2001; Ramos Silva *et al.*, 2002; Paixão *et al.*, 2002) we present here the title compound ethyl 3,5-dimethyl-1*H*-pyrrole-2-carboxylate, (I), Fig. 1. There are two independent molecules per asymmetric unit. The two molecules are very similar and almost planar with the angle between molecular planes being 3.87 (5)°. The molecules show an eclipsed conformation, when viewed along the C1—C7 direction, with the ethoxycarbonylgroup anti to the pyrrole N atom. The molecules are grouped in dimers by strong hydrogen bonds between N—H groups and carbonyl O atoms (Fig.1, Table 1). The dimers stack in planes approximately 5 Å apart (Fig.2).

### Experimental

The ethyl 3,5-dimethyl-1*H*-pyrrole-2-carboxylate was prepared by a Knorr-type reaction from the condensation of acetylacetone and ethyl oximinoacetoacetate.

### Refinement

All H-atoms were positioned geometrically and refined using a riding model with C—H=0.93 Å, N—H=0.86 Å, *U*_iso_(H)=1.2*U*_eq_(C,*N*).

### Crystal data

**Table:**

C_9_H_13_NO_2_	*Z* = 4
*M**_r_* = 167.20	*F*(000) = 360
Triclinic, *P*1	*D*_x_ = 1.191 Mg m^−^^3^
*a* = 8.1357 (2) Å	Mo *K*α radiation, λ = 0.71073 Å
*b* = 10.5568 (2) Å	Cell parameters from 3873 reflections
*c* = 12.1428 (2) Å	θ = 2.4–23.9°
α = 101.5451 (13)°	µ = 0.08 mm^−^^1^
β = 97.8791 (14)°	*T* = 293 K
γ = 110.4821 (14)°	Prism, colourless
*V* = 932.52 (4) Å^3^	0.25 × 0.20 × 0.15 mm

### Data collection

**Table:**

Bruker APEX CCD area-detector diffractometer	4456 independent reflections
Radiation source: fine-focus sealed tube	2368 reflections with *I* > 2σ(*I*)
graphite	*R*_int_ = 0.028
φ and ω scans	θ_max_ = 28.0°, θ_min_ = 1.8°
Absorption correction: multi-scan (SADABS; Sheldrick, 2000)	*h* = −10→10
*T*_min_ = 0.899, *T*_max_ = 0.987	*k* = −13→13
20370 measured reflections	*l* = −15→15

### Refinement

**Table:**

Refinement on *F*^2^	Primary atom site location: structure-invariant direct methods
Least-squares matrix: full	Secondary atom site location: difference Fourier map
*R*[*F*^2^ > 2σ(*F*^2^)] = 0.052	Hydrogen site location: inferred from neighbouring sites
*wR*(*F*^2^) = 0.182	H-atom parameters constrained
*S* = 1.03	*w* = 1/[σ^2^(*F*_o_^2^) + (0.0939*P*)^2^ + 0.0508*P*] where *P* = (*F*_o_^2^ + 2*F*_c_^2^)/3
4456 reflections	(Δ/σ)_max_ < 0.001
223 parameters	Δρ_max_ = 0.20 e Å^−^^3^
0 restraints	Δρ_min_ = −0.21 e Å^−^^3^

### Special details

**Table:**

Geometry. All e.s.d.'s (except the e.s.d. in the dihedral angle between two l.s. planes) are estimated using the full covariance matrix. The cell e.s.d.'s are taken into account individually in the estimation of e.s.d.'s in distances, angles and torsion angles; correlations between e.s.d.'s in cell parameters are only used when they are defined by crystal symmetry. An approximate (isotropic) treatment of cell e.s.d.'s is used for estimating e.s.d.'s involving l.s. planes.
Refinement. Refinement of *F*^2^ against ALL reflections. The weighted *R*-factor *wR* and goodness of fit *S* are based on *F*^2^, conventional *R*-factors *R* are based on *F*, with *F* set to zero for negative *F*^2^. The threshold expression of *F*^2^ > σ(*F*^2^) is used only for calculating *R*-factors(gt) *etc*. and is not relevant to the choice of reflections for refinement. *R*-factors based on *F*^2^ are statistically about twice as large as those based on *F*, and *R*- factors based on ALL data will be even larger.

### Fractional atomic coordinates and isotropic or equivalent isotropic displacement parameters (Å^2^)

**Table:**

	*x*	*y*	*z*	*U*_iso_*/*U*_eq_	
N1	0.2122 (2)	0.18401 (15)	0.96119 (12)	0.0526 (4)	
H1	0.1472	0.1689	0.8941	0.063*	
C1	0.2894 (2)	0.31179 (18)	1.04326 (14)	0.0483 (4)	
C2	0.3837 (3)	0.29357 (19)	1.13867 (15)	0.0535 (5)	
C3	0.3587 (3)	0.1515 (2)	1.11083 (17)	0.0620 (5)	
H3	0.4060	0.1083	1.1589	0.074*	
C4	0.2537 (3)	0.08658 (19)	1.00165 (17)	0.0561 (5)	
C5	0.1877 (3)	−0.0621 (2)	0.92988 (19)	0.0745 (6)	
H5A	0.0653	−0.0912	0.8891	0.112*	
H5B	0.1938	−0.1218	0.9790	0.112*	
H5C	0.2617	−0.0684	0.8756	0.112*	
C6	0.4927 (3)	0.4029 (2)	1.24868 (16)	0.0700 (6)	
H6A	0.5941	0.4716	1.2331	0.105*	
H6B	0.5346	0.3594	1.3024	0.105*	
H6C	0.4193	0.4476	1.2810	0.105*	
C7	0.2626 (2)	0.43124 (19)	1.01696 (15)	0.0497 (4)	
C8	0.3246 (3)	0.67311 (19)	1.08651 (16)	0.0598 (5)	
H8A	0.1987	0.6598	1.0744	0.072*	
H8B	0.3685	0.6956	1.0196	0.072*	
C9	0.4325 (3)	0.7891 (2)	1.19379 (19)	0.0736 (6)	
H9A	0.3901	0.7642	1.2595	0.110*	
H9B	0.4191	0.8742	1.1869	0.110*	
H9C	0.5573	0.8030	1.2035	0.110*	
O1	0.34589 (17)	0.54785 (12)	1.10374 (10)	0.0571 (4)	
O2	0.1739 (2)	0.42886 (14)	0.92727 (11)	0.0697 (4)	
N2	−0.03335 (19)	0.33131 (15)	0.69754 (12)	0.0514 (4)	
H2	0.0341	0.3455	0.7635	0.062*	
C10	−0.0692 (3)	0.43124 (19)	0.65748 (16)	0.0532 (5)	
C11	−0.1793 (3)	0.3672 (2)	0.54923 (17)	0.0581 (5)	
H11	−0.2237	0.4120	0.5012	0.070*	
C12	−0.2140 (2)	0.22325 (19)	0.52282 (15)	0.0511 (5)	
C13	−0.1210 (2)	0.20295 (18)	0.61697 (14)	0.0470 (4)	
C14	−0.3308 (3)	0.1161 (2)	0.41335 (16)	0.0666 (6)	
H14A	−0.2676	0.0606	0.3829	0.100*	
H14B	−0.3600	0.1628	0.3580	0.100*	
H14C	−0.4397	0.0564	0.4290	0.100*	
C15	0.0049 (3)	0.58075 (19)	0.72855 (18)	0.0681 (6)	
H15A	−0.0667	0.5910	0.7837	0.102*	
H15B	0.0018	0.6407	0.6791	0.102*	
H15C	0.1270	0.6063	0.7684	0.102*	
C16	−0.1000 (3)	0.08222 (19)	0.64427 (15)	0.0525 (5)	
C17	−0.1825 (3)	−0.16323 (19)	0.58381 (18)	0.0635 (5)	
H17A	−0.2223	−0.1772	0.6538	0.076*	
H17B	−0.0591	−0.1565	0.5933	0.076*	
C18	−0.3012 (3)	−0.2831 (2)	0.4826 (2)	0.0777 (7)	
H18A	−0.4226	−0.2880	0.4734	0.116*	
H18B	−0.2979	−0.3691	0.4953	0.116*	
H18C	−0.2593	−0.2690	0.4141	0.116*	
O3	−0.19388 (17)	−0.03673 (13)	0.56183 (10)	0.0574 (4)	
O4	−0.0063 (2)	0.08520 (14)	0.73228 (12)	0.0806 (5)	

### Atomic displacement parameters (Å^2^)

**Table:**

	*U*^11^	*U*^22^	*U*^33^	*U*^12^	*U*^13^	*U*^23^
N1	0.0640 (10)	0.0477 (9)	0.0397 (8)	0.0212 (8)	0.0025 (7)	0.0053 (7)
C1	0.0532 (10)	0.0434 (10)	0.0407 (9)	0.0155 (8)	0.0037 (8)	0.0055 (8)
C2	0.0575 (11)	0.0533 (11)	0.0445 (10)	0.0198 (9)	0.0041 (8)	0.0099 (8)
C3	0.0759 (14)	0.0568 (12)	0.0565 (12)	0.0307 (11)	0.0067 (10)	0.0189 (10)
C4	0.0674 (12)	0.0478 (11)	0.0552 (11)	0.0256 (9)	0.0123 (9)	0.0133 (9)
C5	0.0935 (16)	0.0467 (12)	0.0772 (15)	0.0282 (11)	0.0119 (12)	0.0069 (10)
C6	0.0797 (15)	0.0690 (14)	0.0472 (11)	0.0236 (11)	−0.0088 (10)	0.0103 (10)
C7	0.0534 (11)	0.0470 (11)	0.0408 (10)	0.0160 (8)	0.0037 (8)	0.0059 (8)
C8	0.0741 (13)	0.0482 (11)	0.0566 (12)	0.0256 (10)	0.0108 (10)	0.0124 (9)
C9	0.0911 (16)	0.0509 (12)	0.0669 (14)	0.0229 (11)	0.0142 (12)	0.0014 (10)
O1	0.0726 (9)	0.0436 (7)	0.0455 (7)	0.0207 (6)	−0.0015 (6)	0.0050 (6)
O2	0.0918 (11)	0.0559 (8)	0.0498 (8)	0.0314 (8)	−0.0130 (7)	0.0039 (6)
N2	0.0561 (9)	0.0459 (9)	0.0428 (8)	0.0157 (7)	0.0011 (7)	0.0061 (7)
C10	0.0583 (11)	0.0456 (11)	0.0535 (11)	0.0191 (9)	0.0097 (9)	0.0126 (9)
C11	0.0639 (12)	0.0531 (12)	0.0538 (11)	0.0222 (9)	0.0002 (9)	0.0166 (9)
C12	0.0511 (10)	0.0515 (11)	0.0439 (10)	0.0166 (9)	0.0035 (8)	0.0089 (8)
C13	0.0496 (10)	0.0423 (10)	0.0403 (9)	0.0133 (8)	0.0035 (8)	0.0050 (7)
C14	0.0691 (13)	0.0649 (13)	0.0491 (11)	0.0186 (11)	−0.0086 (10)	0.0081 (10)
C15	0.0796 (15)	0.0451 (12)	0.0702 (13)	0.0218 (10)	0.0066 (11)	0.0068 (10)
C16	0.0580 (11)	0.0476 (11)	0.0426 (10)	0.0169 (9)	0.0017 (9)	0.0050 (8)
C17	0.0701 (13)	0.0484 (12)	0.0643 (13)	0.0226 (10)	0.0037 (10)	0.0073 (10)
C18	0.0850 (16)	0.0475 (12)	0.0825 (16)	0.0204 (11)	0.0047 (12)	−0.0019 (11)
O3	0.0664 (8)	0.0428 (7)	0.0508 (8)	0.0179 (6)	−0.0027 (6)	0.0029 (6)
O4	0.1088 (12)	0.0548 (9)	0.0572 (9)	0.0290 (8)	−0.0237 (8)	0.0037 (7)

### Geometric parameters (Å, °)

**Table:**

N1—C4	1.346 (2)	N2—C10	1.348 (2)
N1—C1	1.380 (2)	N2—C13	1.380 (2)
N1—H1	0.8600	N2—H2	0.8600
C1—C2	1.384 (2)	C10—C11	1.373 (3)
C1—C7	1.440 (2)	C10—C15	1.498 (2)
C2—C3	1.404 (3)	C11—C12	1.405 (2)
C2—C6	1.498 (3)	C11—H11	0.9300
C3—C4	1.369 (3)	C12—C13	1.378 (2)
C3—H3	0.9300	C12—C14	1.500 (2)
C4—C5	1.498 (3)	C13—C16	1.438 (3)
C5—H5A	0.9600	C14—H14A	0.9600
C5—H5B	0.9600	C14—H14B	0.9600
C5—H5C	0.9600	C14—H14C	0.9600
C6—H6A	0.9600	C15—H15A	0.9600
C6—H6B	0.9600	C15—H15B	0.9600
C6—H6C	0.9600	C15—H15C	0.9600
C7—O2	1.212 (2)	C16—O4	1.213 (2)
C7—O1	1.336 (2)	C16—O3	1.333 (2)
C8—O1	1.443 (2)	C17—O3	1.444 (2)
C8—C9	1.504 (3)	C17—C18	1.497 (3)
C8—H8A	0.9700	C17—H17A	0.9700
C8—H8B	0.9700	C17—H17B	0.9700
C9—H9A	0.9600	C18—H18A	0.9600
C9—H9B	0.9600	C18—H18B	0.9600
C9—H9C	0.9600	C18—H18C	0.9600
			
C4—N1—C1	109.98 (15)	C10—N2—C13	109.89 (15)
C4—N1—H1	125.0	C10—N2—H2	125.1
C1—N1—H1	125.0	C13—N2—H2	125.1
N1—C1—C2	107.62 (15)	N2—C10—C11	107.26 (16)
N1—C1—C7	119.00 (15)	N2—C10—C15	121.47 (17)
C2—C1—C7	133.38 (16)	C11—C10—C15	131.26 (18)
C1—C2—C3	105.90 (16)	C10—C11—C12	108.90 (17)
C1—C2—C6	127.36 (17)	C10—C11—H11	125.6
C3—C2—C6	126.73 (17)	C12—C11—H11	125.6
C4—C3—C2	109.18 (17)	C13—C12—C11	106.15 (16)
C4—C3—H3	125.4	C13—C12—C14	128.26 (17)
C2—C3—H3	125.4	C11—C12—C14	125.59 (17)
N1—C4—C3	107.32 (16)	C12—C13—N2	107.79 (15)
N1—C4—C5	121.31 (18)	C12—C13—C16	133.95 (16)
C3—C4—C5	131.37 (19)	N2—C13—C16	118.26 (15)
C4—C5—H5A	109.5	C12—C14—H14A	109.5
C4—C5—H5B	109.5	C12—C14—H14B	109.5
H5A—C5—H5B	109.5	H14A—C14—H14B	109.5
C4—C5—H5C	109.5	C12—C14—H14C	109.5
H5A—C5—H5C	109.5	H14A—C14—H14C	109.5
H5B—C5—H5C	109.5	H14B—C14—H14C	109.5
C2—C6—H6A	109.5	C10—C15—H15A	109.5
C2—C6—H6B	109.5	C10—C15—H15B	109.5
H6A—C6—H6B	109.5	H15A—C15—H15B	109.5
C2—C6—H6C	109.5	C10—C15—H15C	109.5
H6A—C6—H6C	109.5	H15A—C15—H15C	109.5
H6B—C6—H6C	109.5	H15B—C15—H15C	109.5
O2—C7—O1	122.56 (16)	O4—C16—O3	121.97 (17)
O2—C7—C1	124.92 (16)	O4—C16—C13	124.71 (16)
O1—C7—C1	112.52 (15)	O3—C16—C13	113.32 (15)
O1—C8—C9	106.73 (15)	O3—C17—C18	107.56 (16)
O1—C8—H8A	110.4	O3—C17—H17A	110.2
C9—C8—H8A	110.4	C18—C17—H17A	110.2
O1—C8—H8B	110.4	O3—C17—H17B	110.2
C9—C8—H8B	110.4	C18—C17—H17B	110.2
H8A—C8—H8B	108.6	H17A—C17—H17B	108.5
C8—C9—H9A	109.5	C17—C18—H18A	109.5
C8—C9—H9B	109.5	C17—C18—H18B	109.5
H9A—C9—H9B	109.5	H18A—C18—H18B	109.5
C8—C9—H9C	109.5	C17—C18—H18C	109.5
H9A—C9—H9C	109.5	H18A—C18—H18C	109.5
H9B—C9—H9C	109.5	H18B—C18—H18C	109.5
C7—O1—C8	116.76 (13)	C16—O3—C17	116.64 (14)
			
C4—N1—C1—C2	0.3 (2)	C13—N2—C10—C11	−0.8 (2)
C4—N1—C1—C7	179.58 (15)	C13—N2—C10—C15	178.44 (16)
N1—C1—C2—C3	−0.4 (2)	N2—C10—C11—C12	0.8 (2)
C7—C1—C2—C3	−179.57 (19)	C15—C10—C11—C12	−178.28 (19)
N1—C1—C2—C6	178.72 (18)	C10—C11—C12—C13	−0.6 (2)
C7—C1—C2—C6	−0.4 (3)	C10—C11—C12—C14	179.03 (18)
C1—C2—C3—C4	0.4 (2)	C11—C12—C13—N2	0.1 (2)
C6—C2—C3—C4	−178.74 (19)	C14—C12—C13—N2	−179.49 (17)
C1—N1—C4—C3	0.0 (2)	C11—C12—C13—C16	−179.8 (2)
C1—N1—C4—C5	−179.76 (16)	C14—C12—C13—C16	0.6 (3)
C2—C3—C4—N1	−0.3 (2)	C10—N2—C13—C12	0.4 (2)
C2—C3—C4—C5	179.4 (2)	C10—N2—C13—C16	−179.68 (15)
N1—C1—C7—O2	1.0 (3)	C12—C13—C16—O4	177.7 (2)
C2—C1—C7—O2	−179.9 (2)	N2—C13—C16—O4	−2.2 (3)
N1—C1—C7—O1	−179.56 (14)	C12—C13—C16—O3	−1.7 (3)
C2—C1—C7—O1	−0.5 (3)	N2—C13—C16—O3	178.41 (15)
O2—C7—O1—C8	0.4 (3)	O4—C16—O3—C17	1.7 (3)
C1—C7—O1—C8	−178.98 (15)	C13—C16—O3—C17	−178.86 (15)
C9—C8—O1—C7	−178.86 (15)	C18—C17—O3—C16	178.37 (16)

### Hydrogen-bond geometry (Å, °)

**Table:**

*D*—H···*A*	*D*—H	H···*A*	*D*···*A*	*D*—H···*A*
N1—H1···O4	0.86	2.02	2.857 (2)	166.
N2—H2···O2	0.86	2.00	2.834 (2)	163.
